# A population‐based study of age‐related associations between vaginal pH and the development of cervical intraepithelial neoplasia

**DOI:** 10.1002/cam4.2845

**Published:** 2020-01-14

**Authors:** Peng Teng, Min Hao

**Affiliations:** ^1^ Department of Obstetrics and Gynecology Second Hospital of Shanxi Medical University Taiyuan China

**Keywords:** cervical cancer, cervical intraepithelial neoplasia, human papillomavirus, vaginal pH

## Abstract

The association between vaginal pH and the risk of cervical intraepithelial neoplasia (CIN) is unclear. We evaluated the dose‐response relationship between vaginal pH and CIN risk, as well as the combined influence of vaginal pH and high‐risk human papillomavirus (hrHPV) on the risk of CIN and the mediation effects of hrHPV infection on vaginal pH level and the development of CIN. We investigated 2304 women in Shanxi, China. The dose‐response relationship between vaginal pH and CIN risk was assessed using categoric and spline analyses. We established interaction and mediation models to determine the correlation between pH and hrHPV in the development of CIN. After adjusting covariates, a positive association was observed between hrHPV infection and the development of CIN [OR (95% CI) = 4.75 (3.52‐6.40) for CIN2+; OR (95% CI) = 7.30 (4.10‐13.00) for CIN3+], while a negative correlation was showed between vaginal pH level and CIN3+ [OR (95% CI) = 1.04 (0.59‐1.84); high vs low: OR (95% CI) = 0.32 (0.15‐0.69), *P* = .002]. The highest risk of CIN (5.24 of CIN2+ and 5.80 of CIN3+) were observed when hrHPV infection was combined with middle vaginal pH (4.6‐5.0). A significant mediation effect of hrHPV infection was observed in the association between vaginal pH level with CIN2+ (*P* = .002) and CIN3+ (*P* = .004). In conclusion, abnormal vaginal pH significantly induced the risk of high‐stage CIN in Chinese women infected with hrHPV. Therefore, maintaining normal vaginal pH levels may reduce the risk of CIN.

## Graphical abstract

Cervical cancer is one of the most common gynecological malignancies among women. We explored the link between vaginal pH, HPV, and cervical intraepithelial neoplasia. CIN risk was highest in hrHPV‐positive patients with middle vaginal pH (4.6‐5.0). Maintaining normal vaginal pH levels may reduce the risk of CIN.

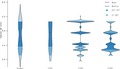

## 1 INTRODUCTION

Cervical cancer is the second most common malignant tumor among women worldwide.^1^ Contrary with the decreasing incidence trends in Western countries,^2^ cervical cancer incidence has substantially increased in China.^3^, ^4^ About 132,000 cases of cervical cancer have occurred in China, accounting for 28% of the world's total cervical cancer burden. Shanxi province located in northern China now has the highest incidence of cervical cancer, with incidence rates being 10 times higher than the national average.^5^ Cervical intraepithelial neoplasia (CIN) is the precancerous lesion of the cervix, which is related to invasive cervical cancer, caused by persistent infection of high‐risk human papillomavirus (hrHPV).^6^, ^7^ The progression from CIN to cervical cancer needs about 8‐12 years, so cervical cancer is a preventable disease.^8^ Risk prediction should be the first step in the prevention of cervical cancer. Population‐based cervical cancer research in several Western countries have been reported. Risk factors for CIN and cervical cancer include socioeconomic status, race, smoking, younger age at first intercourse, high parity, and oral contraceptive use.^9^, ^10^, ^11^ However, relatively little is known about nonbehavioral factors that influence the risk of CIN.

Recent studies have suggested that the occurrence of CIN is related to the lactobacillus in the vaginal microenvironment.^12^, ^13^ The vaginal microenvironment, such as the presence of lactobacilli and an acidic vaginal pH, are critical components of the vaginal defense system.^13^ Vaginal Lactobacillus can maintain and activate the immune function by holding an acidic environment in the vagina, which can reduce the incidence of cervical cancer.^14^, ^15^ A few recent case‐control studies of squamous intraepithelial lesions (SIL) or CIN, including a relatively small number of subjects with high grade squamous intraepithelial lesions (HSIL) or CIN3, have prompted that the elevated vaginal pH is related to the detection of HPV and SIL in certain age groups after adjusting for the effect of HPV.^16^ Although the mechanisms for these associations are not entirely understood, vaginal pH may be able to become a risk predictor relating to CIN.

In healthy, reproductive‐aged women, vaginal pH is primarily determined by the lactic acid produced by the metabolically active epithelium and from Lactobacillus species that dominate the vaginal microflora and produce lactic acid from anaerobic glycolysis.^17^ A vaginal pH range of 3.8‐4.5 is considered normal for premenopausal women.^18^ After menopause, this rise in vaginal pH is associated with a loss of natural epithelial defenses.^19^ A large‐scale population‐based study has confirmed that vaginal pH rises with age, starting in the peri‐menopausal age range.^20^ Besides, the risk of HPV infection may be exacerbated by an age‐related attenuation of the immune response and reductions of the natural immune defenses of the skin, typical of menopause.^20^, ^21^ However, there have been limited population‐based studies on age‐related vaginal pH, and the risk of CIN and previous studies have been severely limited by a lack of control for HPV infection. The firm association between age‐related vaginal pH and the risk of CIN is warranted to investigate.

Therefore, our study estimated the relationship between vaginal pH change and the risk of CIN, with attention to possible differences between women in varying age groups in Shanxi in 2014 (Shanxi CIN Cohort), and purposed to reduce cervical cancer incidence and mortality.

## 2 MATERIALS AND METHODS

### 2.1 Study participants

Between June 2014 and December 2014, 40,000 women aged 19‐65 years who were long‐term (≥5 years) residents of the Yangqu and Jiexiu regions of Shanxi Province completed questionnaires and underwent examinations for vaginal pH, HPV, and liquid‐based cytology (LBC). Colposcopy and histopathologic analysis were performed using LBC for atypical squamous cells of undetermined significance (ASC‐US) and above stages. All processes were implemented under double‐blind conditions. Among 2769 women diagnosed with ASC‐US or above, 10 were excluded for cervical gland cell abnormalities, and 68 refused a biopsy. Among 1890 patients with negative cervical biopsy results, 387 were excluded owing to incomplete data. A final total of 2304 patients were analyzed; these included 1503 who were CIN‐negative, 564 with CIN1, 171 with CIN2, 47 with CIN3, and 19 with squamous cell carcinoma (SCC). The study was approved by the Ethics Committee of our institution; all participants provided written informed consent. A standardized structured questionnaire was administered to each participant through an in‐person interview to collect information on demographic factors, smoking and alcohol consumption, menstrual, maternal, and other medical histories.

### 2.2 Demographic characteristics and identifying factors related to cervical lesions

Face‐to‐face interviews were conducted by qualified investigators using standardized questionnaires that collected demographic data; alcohol drinking and smoking habits; and menstrual, maternal, and other medical histories.

### 2.3 Liquid‐based cytology

All subjects were required to abstain from sex, vaginal lavages, and medications for 48 hours. Pap smear detection was performed using LBC (Lituo Biotechnology). Two cytopathologists evaluated according to the revised Bethesda system (2001). ASC‐US and higher‐stage lesions were reviewed by senior physicians who were unaware of the original pathological findings.

### 2.4 HPV genotyping

Genotyping was performed using a flow‐through hybridization kit (HybriBio Ltd) that can detect 21 types of HPV including 15 high‐risk types (16, 18, 31, 33, 35, 39, 45, 51, 52, 53, 56, 58, 59, 66, and 68) and six low‐risk types (6, 11, 41, 42, 44, and 81).

### 2.5 Colposcopy and histopathology of cervical tissue

Within 12 weeks after the Pap smear examination, a gynecologist at our hospital performed a colposcopy (SLC‐2000 device; Shenzhen Goldway Company). The cervix was divided into four quadrants for testing, and all visible abnormalities were biopsied; if no visible lesions were present, random biopsies were acquired at the junctions of each quadrant. Endocervical curettage was performed in patients with abnormal cytology but with negative or unsatisfactory colposcopy. Cervical biopsy and endocervical curettage specimens were evaluated by two gynecologists. Pathological results were classified as negative, CIN1, CIN2, CIN3, and SCC. Pathological sections were reviewed by two pathologists who were blinded to the cytology and HPV results; disagreements were arbitrated by a senior pathologist to obtain consensus.

### 2.6 Vaginal pH detection

All participants were instructed to abstain from sexual intercourse and not perform vaginal lavage or take medications for 48 hours prior to the sampling. Vaginal pH levels were measured and scored using Vaginitis Multi‐Test kits (Drying Chemoenzymatic Method; Bioperfectus Technologies) according to the manufacturer's instructions.

### 2.7 Statistical methods

The Pearson chi‐square test and Kruskal‐Wallis H test were respectively used for categorical variables and numerical variables. The associations of vaginal pH levels with CIN were estimated using the multivariate logistic regression models. A spline analysis was applied to examine the dose‐response relationship between vaginal pH and the risk of CIN. A crossover model and a mediation model were respectively used to evaluate the interaction between vaginal pH level and hrHPV infection, and whether vaginal pH has a mediating effect in CIN caused by hrHPV, both models were adjusted for age, education, occupation, yearly family income, smoking status, drinking status, and clean the vagina. Smoking was defined as those who smoked at least 1 cigarette/d in the past 6 months. Drinking was defined as those who drank hard liquor, beer, or wine at least 1/wk in the past 6 months. Statistical analyses were performed using the SAS software version 9.4 (SAS Institute Inc). All reported *P*‐values are based on 2‐sided tests; *P* < .05 is considered significant.

## 3 RESULTS

### 3.1 Main characteristics of study participants

2304 were included in this study, whose age at the time of visit range from 19 to 66 years. The main features of study participants by vaginal pH level were summarized in Table 1. Women with high vaginal pH were older [55 (50‐59) years], had lower yearly family income, while less likely to have high‐risk HPV infection than those low‐pH women. There were no significances in smoking status, drinking status, and clean the vagina (*P* > .05).

**Table 1:** Basic characteristics of 2304 Chinese participants by vaginal pH level^a^

Variable	Vaginal pH level				*P*‐value^b^
	Total (n = 2304)	Low (≤4.5) (n = 732)	Middle (4.6‐5.0) (n = 722)	High (>5.0) (n = 850)	
General characteristic					
Age (y)	50 (43‐56)	47 (41‐52)	48 (41‐54)	55 (50‐59)	<.001
Education (y)					
1‐9	1466 (63.5)	457 (61.9)	464 (63.9)	545 (64.3)	.44
10‐12	520 (22.7)	161 (22.3)	159 (22.2)	200 (23.4)	
>12	318 (13.8)	114 (15.8)	99 (13.9)	105 (12.3)	
Occupation					
Office worker	367 (15.3)	136 (17.8)	118 (15.8)	113 (13.1)	<.001
Farmer	741 (33.4)	191 (27.2)	224 (32.1)	326 (39.1)	
Other	409 (17.4)	150 (20.3)	120 (16.4)	139 (16.1)	
Unemployed	787 (33.9)	255 (34.7)	260 (35.8)	272 (31.7)	
Yearly family income (RMB)					
≤10 000	744 (34.2)	206 (29.5)	191 (28.6)	347 (41.8)	<.001
10 000‐20 000	610 (26.5)	203 (27.8)	195 (27.2)	212 (25)	
20 000‐30 000	474 (19.9)	147 (19.7)	164 (21.9)	163 (18.5)	
>30 000	476 (19.5)	176 (23)	172 (22.2)	128 (14.6)	
Smoking status					
No	2254 (97.6)	722 (98.4)	703 (97.2)	829 (97.3)	.191
Yes	50 (2.4)	10 (1.6)	19 (2.8)	21 (2.7)	
Drinking status					
No	2219 (96.3)	701 (95.8)	698 (96.7)	820 (96.5)	.623
Yes	85 (3.7)	31 (4.2)	24 (3.3)	30 (3.5)	
Clean the vagina					
No	470 (21.2)	140 (20)	146 (21)	184 (22.3)	.458
Yes	1834 (78.8)	592 (80)	576 (79)	666 (77.7)	
Laboratory examination					
High‐risk HPV infection					
No	1552 (68.6)	445 (61.8)	469 (65.8)	638 (76)	<.001
Yes	752 (31.4)	287 (38.2)	253 (34.2)	212 (24)	
Pathological type					
Normal	1503 (65.6)	480 (66.3)	457 (63.4)	566 (66.6)	.003
CIN1	564 (24.5)	164 (21.9)	177 (24.6)	223 (26.5)	
CIN2	171 (7.0)	59 (7.5)	61 (8.2)	51 (5.8)	
CIN3+^c^	66 (2.9)	29 (4.3)	27 (3.9)	10 (1.1)	

a *P*‐values were calculated from the Kruskal‐Wallis H test for numerical variables.

b *P*‐values were calculated from the Chi‐square test for categorical variables.

c CIN3+: CIN3 and cervical cancer.

**Figure 1:**
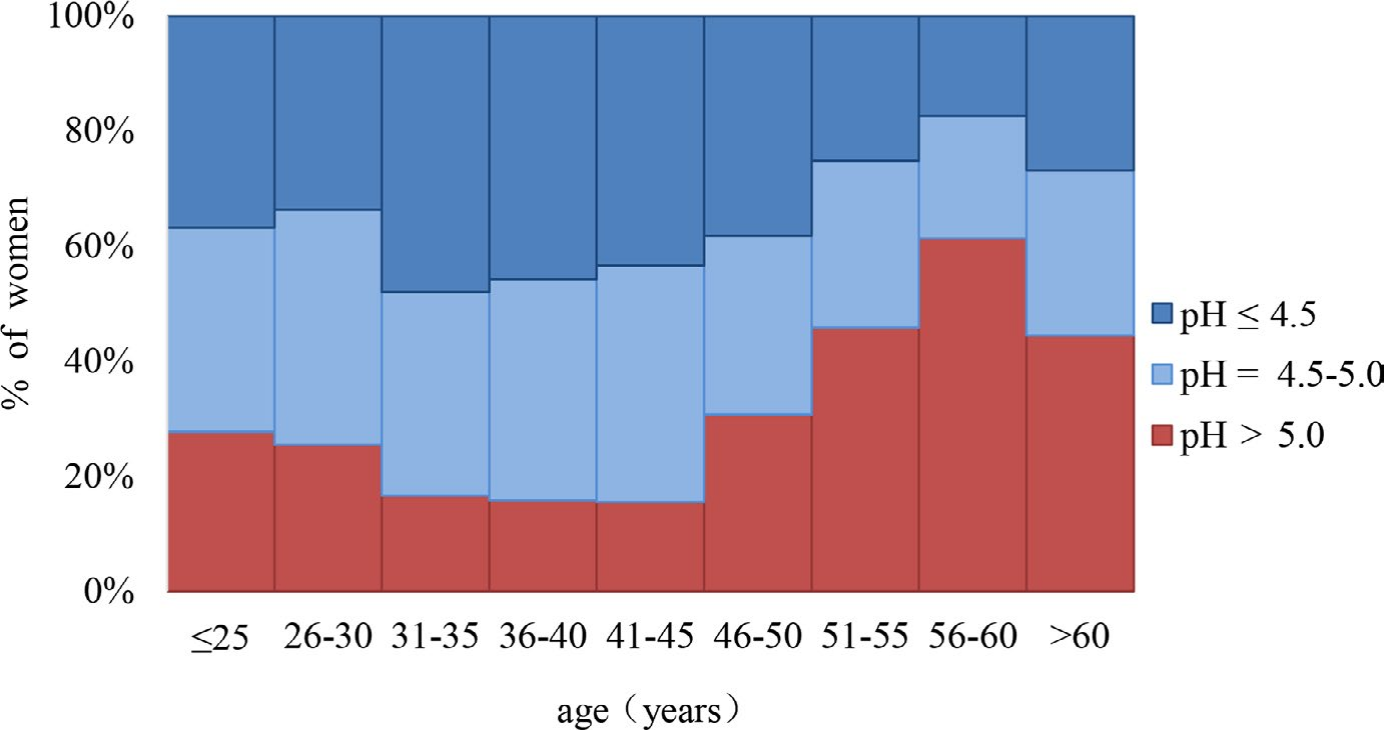
The percentage of women with a given pH measurement stratified by age. The dark blue indicates the percentage of women with a vaginal pH of ≤4.5, the blue shows the percentage of women with a vaginal pH of 4.6‐5.0, and the red indicates the percentage of women with a vaginal pH of >5.0

**Figure 2:**
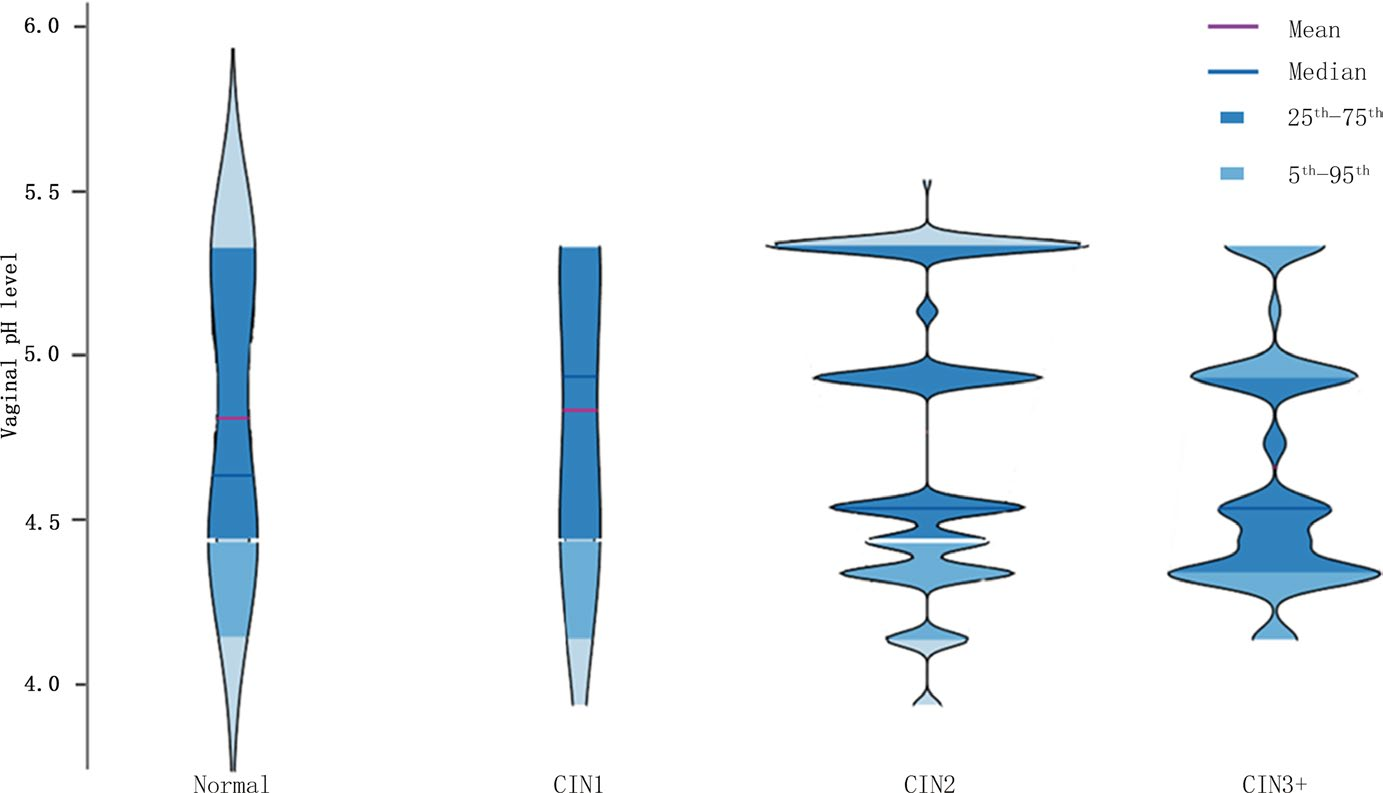
The distributions of vaginal pH level in different CIN pathological types. A violin plot showed the vaginal pH level in different CIN pathological types. *P*‐values were calculated from the Kruskal‐Wallis H test. The red line represents mean, the blue line represents the median, the dark blue indicates the range from the 25th percentile to 75th percentile, and the blue shows the field from the 5th percentile to 95th percentile

Figure 1 shows the percentage of women with a vaginal pH constitution by age. The majority of younger women (≤50 years old) had a pH ≤ 4.5 (29.4%‐34.5% for each age group) while a relatively low percentage had a pH > 5.0 (12.5%‐27.5%). On the other hand, almost half of older women (>50 years old) had a pH of at least 5.0 (43.1%‐60.3%) and a relatively higher percentage of these age groups compared to younger women. Besides, the detailed distributions of vaginal pH levels in different CIN pathological types were given in Figure 2 with a violin plot. During the four CIN pathological types, the median of vaginal pH level rose in advance then fell with the development of CIN (*P* < .05).

### 3.2 Effects of high‐risk HPV infection or vaginal pH level on the development of CIN

The odds ratios (ORs) and 95% confidence intervals (CIs) for the effects of high‐risk HPV infection or vaginal pH levels on the development of CIN were presented in Table 2. After adjusting covariates, a positive association was showed between high‐risk HPV infection and the development of CIN [OR (95% CI) = 4.03 (2.87‐5.66) for CIN2; OR (95% CI) = 4.75 (3.52‐6.40) for CIN2+; OR (95% CI) = 7.30 (4.10‐13.00) for CIN3+], while a negative association was showed between vaginal pH level and CIN3+ [OR (95% CI) = 1.04 (0.59‐1.84); high vs low: OR (95% CI) = 0.32 (0.15‐0.69), *P* = .002]. The significant effects of vaginal pH level on CIN3+ were also observed in older (age > 50) women [OR (95% CI) = 0.20 (0.07‐0.63); high vs middle pH: OR (95% CI) = 0.72(0.28‐1.86), *P* = .005]. However, the effects of vaginal pH level on the development of CIN were not significant in other age women (*P* > .05).

**Table 2:** Effects of high‐risk HPV infection or vaginal pH level on the development of CIN

	n	OR (95% CI)			
		Unadjusted	Model 1^a^	Model 2^b^	Model 3^c^
*CIN1*					
All women					
HrHPV infection	564/1503	1.06 (0.85‐1.31)	1.08 (0.87‐1.34)	1.05 (0.85‐1.30)	11.07 (0.86‐1.33)
Vaginal pH level^d^					
Low pH	164/480	1.00 (reference)	1.00 (reference)	1.00 (reference)	1.00 (reference)
Middle pH	177/457	1.13 (0.89‐1.45)	1.11 (0.86‐1.42)	1.14 (0.89‐1.46)	1.11 (0.86‐1.43)
High pH	223/566	1.15 (0.91‐1.46)	1.15 (0.90‐1.46)	1.18 (0.92‐1.51)	1.16 (0.90‐1.49)
*P*‐trend		.344	.356	.262	.319
Age ≤50					
HrHPV infection	281/731	1.12 (0.84‐1.48)	1.17 (0.87‐1.57)	1.12 (0.84‐1.48)	1.17 (0.87‐1.57)
Vaginal pH level					
Low pH	114/304	1.00 (reference)	1.00 (reference)	1.00 (reference)	1.00 (reference)
Middle pH	110/265	1.11 (0.81‐1.51)	1.05 (0.76‐1.44)	1.11 (0.81‐1.51)	1.05 (0.76‐1.44)
High pH	57/162	0.94 (0.65‐1.36)	0.91 (0.63‐1.34)	0.94 (0.65‐1.37)	0.92 (0.63‐1.34)
*P*‐trend		.630	.584	.658	.591
Age >50					
HrHPV infection	283/772	0.96 (0.69‐1.34)	0.95 (0.67‐1.33)	0.96 (0.69‐1.34)	0.95 (0.67‐1.33)
Vaginal pH level					
Low pH	50/176	1.00 (reference)	1.00 (reference)	1.00 (reference)	1.00 (reference)
Middle pH	67/192	1.23 (0.81‐1.87)	1.20 (0.79‐1.84)	1.23 (0.81‐1.86)	1.20 (0.78‐1.84)
High pH	166/404	1.45 (1.01‐2.08)	1.38 (0.96‐2.00)	1.42 (0.99‐2.05)	1.37 (0.94‐1.98)
*P*‐trend		.052	.096	.072	.118
*CIN2*					
All women					
HrHPV infection	171/1503	4.31 (3.10‐5.99)	4.26 (3.05‐5.96)	4.05 (2.90‐5.66)	4.03 (2.87‐5.66)
Vaginal pH level					
Low pH	59/480	1.00 (reference)	1.00 (reference)	1.00 (reference)	1.00 (reference)
Middle pH	61/457	1.09 (0.74‐1.59)	1.16 (0.78‐1.73)	1.10 (0.75‐1.61)	1.18 (0.79‐1.76)
High pH	51/566	0.73 (0.49‐1.09)	0.84 (0.55‐1.26)	0.89 (0.59‐1.34)	0.95 (0.62‐1.46)
*P*‐trend		.058	.227	.428	.602
Age ≤50					
HrHPV infection	116/731	4.32 (2.82‐6.62)	4.20 (2.70‐6.54)	4.31 (2.81‐6.61)	4.19 (2.69‐6.52)
Vaginal pH level					
Low pH	52/304	1.00 (reference)	1.00 (reference)	1.00 (reference)	1.00 (reference)
Middle pH	46/265	1.02 (0.66‐1.56)	1.15 (0.73‐1.82)	1.01 (0.66‐1.55)	1.15 (0.72‐1.81)
High pH	18/162	0.65 (0.37‐1.15)	0.66 (0.36‐1.20)	0.65 (0.37‐1.15)	0.66 (0.37‐1.20)
*P*‐trend		.119	.134	.126	.14
Age >50					
HrHPV infection	55/772	3.27 (1.88‐5.71)	3.59 (2.02‐6.35)	3.25 (1.86‐5.68)	3.56 (2.01‐6.32)
Vaginal pH level					
Low pH	7/176	1.00 (reference)	1.00 (reference)	1.00 (reference)	1.00 (reference)
Middle pH	15/192	1.96 (0.78‐4.93)	1.93 (0.76‐4.92)	1.95 (0.78‐4.89)	1.90 (0.75‐4.84)
High pH	33/404	2.05 (0.89‐4.73)	2.13 (0.91‐4.99)	1.94 (0.84‐4.5)	1.98 (0.84‐4.67)
*P*‐trend		.198	.165	.272	.251
*CIN2+* e					
All women					
HrHPV infection	237/1503	4.93 (3.68‐6.60)	4.83 (3.59‐6.50)	4.77 (3.56‐6.40)	4.75 (3.52‐6.40)
Vaginal pH level					
Low pH	88/480	1.00 (reference)	1.00 (reference)	1.00 (reference)	1.00 (reference)
Middle pH	88/457	1.05 (0.76‐1.45)	1.12 (0.80‐1.57)	1.06 (0.77‐1.46)	1.12 (0.80‐1.58)
High pH	61/566	0.59 (0.42‐0.83)	0.68 (0.47‐0.98)	0.65 (0.46‐0.94)	0.71 (0.49‐1.04)
*P*‐trend		.001	.014	.008	.035
Age ≤50					
HrHPV infection	154/731	4.08 (2.80‐5.93)	4.04 (2.74‐5.95)	4.08 (2.81‐5.94)	4.06 (2.75‐5.99)
Vaginal pH level					
Low pH	67/304	1.00 (reference)	1.00 (reference)	1.00 (reference)	1.00 (reference)
Middle pH	64/265	1.10 (0.75‐1.60)	1.22 (0.81‐1.83)	1.10 (0.75‐1.61)	1.23 (0.81‐1.84)
High pH	23/162	0.64 (0.39‐1.07)	0.65 (0.38‐1.12)	0.64 (0.38‐1.07)	0.65 (0.38‐1.11)
*P*‐trend		.068	.08	.065	.075
Age >50					
HrHPV infection	83/772	5.53 (3.45‐8.87)	5.73 (3.53‐9.31)	5.51 (3.43‐8.84)	5.71 (3.51‐9.28)
Vaginal pH level					
Low pH	21/176	1.00 (reference)	1.00 (reference)	1.00 (reference)	1.00 (reference)
Middle pH	24/192	1.05 (0.56‐1.95)	1.08 (0.56‐2.08)	1.04 (0.56‐1.93)	1.08 (0.56‐2.09)
High pH	38/404	0.79 (0.45‐1.38)	0.91 (0.50‐1.66)	0.74 (0.42‐1.30)	0.85 (0.46‐1.55)
*P*‐trend		.273	.608	.180	.432
*CIN3+* f					
All women					
HrHPV infection	66/1503	7.24 (4.12‐12.71)	6.99 (3.93‐12.41)	7.49 (4.25‐13.21)	7.30 (4.10‐13.00)
Vaginal pH level					
Low pH	29/480	1.00 (Reference)	1.00 (Reference)	1.00 (reference)	1.00 (reference)
Middle pH	27/457	0.98 (0.57‐1.68)	1.03 (0.58‐1.82)	0.97 (0.57‐1.67)	1.04 (0.59‐1.84)
High pH	10/566	0.29 (0.14‐0.61)	0.36 (0.17‐0.76)	0.26 (0.13‐0.56)	0.32 (0.15‐0.69)
*P*‐trend		.001	.005	≤.001	.002
Age ≤50					
HrHPV infection	38/731	3.44 (1.73‐6.84)	3.58 (1.73‐7.43)	3.53 (1.77‐7.04)	3.77 (1.80‐7.90)
Vaginal pH level					
Low pH	15/304	1.00 (reference)	1.00 (reference)	1.00 (reference)	1.00 (reference)
Middle pH	18/265	1.38 (0.68‐2.79)	1.42 (0.66‐3.05)	1.44 (0.71‐2.92)	1.50 (0.69‐3.24)
High pH	5/162	0.63 (0.22‐1.75)	0.64 (0.22‐1.83)	0.59 (0.21‐1.65)	0.61 (0.21‐1.76)
*P*‐trend		.292	.310	.234	.271
Age >50					
HrHPV infection	28/772	21.9 (7.50‐64.01)	19.71 (6.58‐59.03)	21.70 (7.42‐63.44)	19.68 (6.56‐59.01)
Vaginal pH level					
Low pH	14/176	1.00 (reference)	1.00 (reference)	1.00 (reference)	1.00 (reference)
Middle pH	9/192	0.59 (0.25‐1.40)	0.70 (0.27‐1.81)	0.59 (0.25‐1.39)	0.72 (0.28‐1.86)
High pH	5/404	0.16 (0.06‐0.44)	0.22 (0.07‐0.68)	0.14 (0.05‐0.40)	0.20 (0.07‐0.63)
*P*‐trend		.001	.008	≤.001	.005

a Model 1: adjusted for age, education, occupation, yearly family income.

b Model 2: adjusted for age, education, occupation, yearly family income, smoking status, drinking status, and clean the vagina.

c Model 3: adjusted for age, education, occupation, yearly family income, smoking status, drinking status, clean the vagina, vaginal pH, or hrHPV.

d Low pH: vaginal pH level ≤4.5; Middle pH: vaginal pH level = 4.6‐5.0; High pH: vaginal pH level >5.0.

e CIN2+: CIN2, CIN3, and cervical cancer.

f CIN3+: CIN3, and cervical cancer.

**Figure 3:**
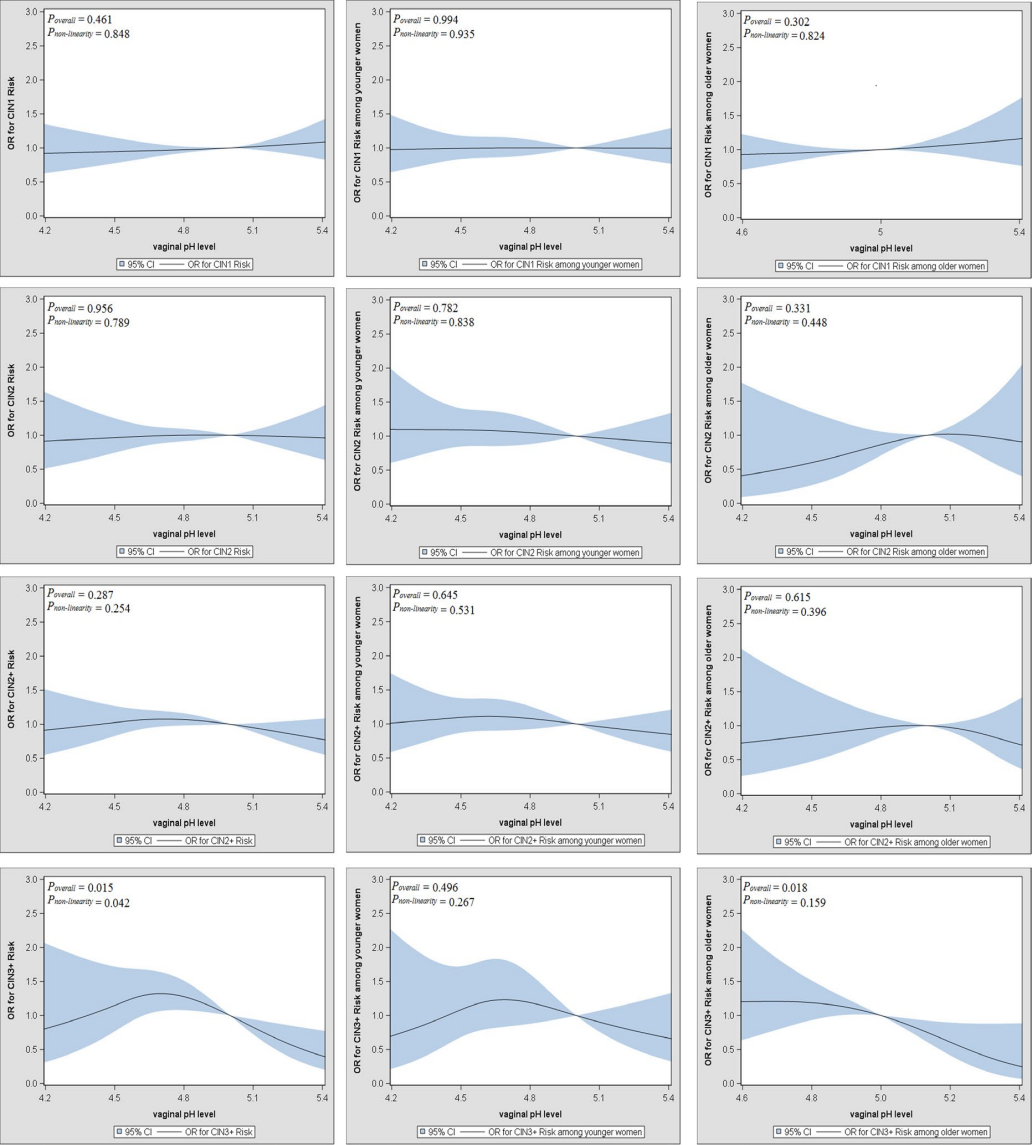
Restricted cubic spline analysis between vaginal pH level and the development of CIN. Data were presented as odds ratio (OR) and 95% confidence interval (95% CI)

Figure 3 depicted the dose‐response relationships between vaginal pH level and the development of CIN, which were performed with 3‐knot (25th, 50th, and 75th percentiles) restricted cubic splines (RCS) functions. Results showed there were nonlinear associations between vaginal pH level and CIN3+ (*P*_overall_ = .015, *P*_non‐linearity_ = .042). After adjusting for covariates, the association between vaginal pH level and CIN3+ were positive up to 4.7 and then fell, while the association between vaginal pH level and other CIN grade (CIN1, CIN2, CIN2+) was not significant (*P* > .05). The consistent results were not observed in different age women.

### 3.3 Interaction effect of high‐risk HPV infection and vaginal pH level on the development of CIN

We explored the interaction effect of high‐risk HPV infection and vaginal pH level on the development of CIN (Table 3). After adjusting covariates, women both with high‐risk HPV infection and a vaginal pH of 4.6‐5.0 had the highest risk of the development of CIN (CIN2, 4.97, CIN2+, 5.24; and CIN3+, 5.80), compared to women without high‐risk HPV infection and a low vaginal pH (≤4.5).

**Table 3:** Interaction effects of high‐risk HPV infection and vaginal pH level on the development of CIN^a^

	High‐risk HPV infection	
	No	Yes
CIN1		
Low pH^b^	1.00 (reference)	1.32 (0.65‐2.67)
Middle pH	1.17 (0.71‐1.93)	1.75 (0.91‐3.38)
High pH	1.60 (1.04‐2.47)	1.00 (0.55‐1.82)
CIN2		
Low pH	1.00 (reference)	3.26 (1.83‐5.79)
Middle pH	0.77 (0.40‐1.50)	4.97 (2.83‐8.76)
High pH	0.95 (0.51‐1.75)	2.93 (1.57‐5.46)
CIN2+^c^		
Low pH	1.00 (reference)	3.98 (2.45‐6.48)
Middle pH	0.81 (0.47‐1.42)	5.24 (3.22‐8.53)
High pH	0.64 (0.37‐1.13)	2.75 (1.60‐4.74)
CIN3+^d^		
Low pH	1.00 (reference)	5.33 (2.27‐12.5)
Middle pH	0.91 (0.33‐2.47)	5.80 (2.44‐13.8)
High pH	0.08 (0.01‐0.63)	2.55 (0.94‐6.94)

a Data were presented as odds ratio (OR) and 95% confidence interval (95% CI); adjusted for age, education, occupation, yearly family income, smoking status, drinking status, and clean the vagina.

b Low pH: vaginal pH level ≤4.5; Middle pH: vaginal pH level = 4.6‐5.0; High pH: vaginal pH level >5.0.

c CIN2+: CIN2, CIN3, and cervical cancer.

d CIN3+: CIN3 and cervical cancer.

### 3.4 Mediation effects of high‐risk HPV infection on vaginal pH level and the development of CIN

To assess whether hrHPV infection could influence the association between vaginal pH level and the development of CIN, we also estimated the effect of vaginal pH level on the hrHPV infection (Table 4). In the all‐women of CIN1, CIN2, CIN2+, and CIN3+, an inverse association between vaginal pH level and hrHPV infection was found, and with the increase in vaginal pH level, the risk of hrHPV infection decreased gradually in four models (*P* < .001). The consistent results were observed in CIN1 and CIN3+ among younger women (≤50 years old).

**Table 4:** Effect of vaginal pH level on high‐risk HPV infection

	OR (95% CI) by vaginal pH level			*P*‐trend
	Low (≤4.5)	Middle (4.6‐5.0)	High (>5.0)	
**High‐risk HPV infection**				
*CIN1*				
All women				
n (yes/no)	228/416	190/440	177/612	
Unadjusted	1.00 (reference)	0.78 (0.62‐0.99)	0.53 (0.42‐0.67)	<.001
Model 1^a^	1.00 (reference)	0.79 (0.63‐1.00)	0.51 (0.41‐0.65)	<.001
Model 2^b^	1.00 (reference)	0.79 (0.63‐1.00)	0.63 (0.50‐0.81)	.001
Model 3^c^	1.00 (reference)	0.81 (0.64‐1.02)	0.62 (0.49‐0.80)	<.001
Age ≤50				
n (yes/no)	171/247	126/249	73/146	
Unadjusted	1.00 (reference)	0.73 (0.55‐0.98)	0.72 (0.51‐1.02)	.114
Model 1	1.00 (reference)	0.76 (0.57‐1.03)	0.69 (0.49‐0.98)	.065
Model 2	1.00 (reference)	0.73 (0.55‐0.97)	0.73 (0.52‐1.03)	.129
Model 3	1.00 (reference)	0.76 (0.56‐1.02)	0.70 (0.49‐1.00)	.079
Age > 50				
n (yes/no)	57/169	64/195	104/466	
Unadjusted	1.00 (reference)	0.97 (0.64‐1.47)	0.66 (0.46‐0.96)	.009
Model 1	1.00 (reference)	0.99 (0.65‐1.50)	0.66 (0.46‐0.96)	.008
Model 2	1.00 (reference)	0.97 (0.64‐1.47)	0.65 (0.45‐0.94)	.007
Model 3	1.00 (reference)	0.99 (0.65‐1.50)	0.65 (0.45‐0.95)	.006
*CIN2*				
All women				
n (yes/no)	202/337	174/344	160/457	
Unadjusted	1.00 (reference)	0.84 (0.66‐1.09)	0.58 (0.46‐0.75)	<.001
Model 1	1.00 (reference)	0.86 (0.66‐1.11)	0.57 (0.44‐0.73)	<.001
Model 2	1.00 (reference)	0.85 (0.66‐1.10)	0.71 (0.55‐0.93)	.017
Model 3	1.00 (reference)	0.88 (0.68‐1.14)	0.70 (0.54‐0.92)	.011
Age ≤50				
n (yes/no)	158/198	120/191	66/114	
Unadjusted	1.00 (reference)	0.79 (0.58‐1.07)	0.73 (0.50‐1.05)	.125
Model 1	1.00 (reference)	0.86 (0.62‐1.18)	0.71 (0.48‐1.03)	.081
Model 2	1.00 (reference)	0.78 (0.57‐1.07)	0.73 (0.51‐1.06)	.138
Model 3	1.00 (reference)	0.85 (0.62‐1.17)	0.71 (0.49‐1.04)	.096
Age >50				
n (yes/no)	44/139	54/153	94/343	
Unadjusted	1.00 (reference)	1.12 (0.70‐1.77)	0.87 (0.58‐1.30)	.253
Model 1	1.00 (reference)	1.13 (0.71‐1.80)	0.88 (0.58‐1.33)	.273
Model 2	1.00 (reference)	1.11 (0.70‐1.76)	0.85 (0.56‐1.28)	.209
Model 3	1.00 (reference)	1.13 (0.71‐1.79)	0.86 (0.57‐1.31)	.241
*CIN2+* d				
All women				
n (yes/no)	223/345	193/352	169/458	
Unadjusted	1.00 (reference)	0.85 (0.67‐1.08)	0.57 (0.45‐0.73)	<.001
Model 1	1.00 (reference)	0.86 (0.67‐1.10)	0.56 (0.44‐0.71)	<.001
Model 2	1.00 (reference)	0.86 (0.67‐1.09)	0.68 (0.52‐0.87)	.003
Model 3	1.00 (reference)	0.87 (0.68‐1.12)	0.66 (0.51‐0.86)	.002
Age ≤50				
n (yes/no)	166/205	132/197	71/114	
Unadjusted	1.00 (reference)	0.83 (0.61‐1.12)	0.77 (0.54‐1.10)	.197
Model 1	1.00 (reference)	0.89 (0.65‐1.21)	0.75 (0.52‐1.09)	.145
Model 2	1.00 (reference)	0.83 (0.61‐1.12)	0.77 (0.54‐1.11)	.207
Model 3	1.00 (reference)	0.89 (0.65‐1.21)	0.76 (0.53‐1.10)	.161
Age >50				
n (yes/no)	57/140	61/155	98/344	
Unadjusted	1.00 (reference)	0.97 (0.63‐1.48)	0.70 (0.48‐1.02)	.032
Model 1	1.00 (reference)	0.96 (0.63‐1.48)	0.70 (0.48‐1.03)	.036
Model 2	1.00 (reference)	0.96 (0.63‐1.48)	0.68 (0.46‐1.00)	.023
Model 3	1.00 (reference)	0.96 (0.62‐1.48)	0.69 (0.46‐1.01)	.027
*CIN3+* e				
All women				
n (yes/no)	185/324	149/335	143/433	
Unadjusted	1.00 (reference)	0.78 (0.60‐1.02)	0.58 (0.45‐0.75)	<.001
Model 1	1.00 (reference)	0.78 (0.60‐1.02)	0.56 (0.43‐0.73)	<.001
Model 2	1.00 (reference)	0.78 (0.60‐1.02)	0.66 (0.51‐0.87)	.007
Model 3	1.00 (reference)	0.79 (0.60‐1.03)	0.65 (0.50‐0.86)	.005
Age ≤50				
n (yes/no)	130/189	98/185	59/108	
Unadjusted	1.00 (reference)	0.77 (0.55‐1.07)	0.79 (0.54‐1.17)	.346
Model 1	1.00 (reference)	0.82 (0.58‐1.16)	0.78 (0.53‐1.17)	.288
Model 2	1.00 (reference)	0.77 (0.55‐1.07)	0.80(0.54‐1.18)	.362
Model 3	1.00 (reference)	0.82 (0.58‐1.15)	0.79 (0.53‐1.18)	.320
Age >50				
n (yes/no)	55/135	51/150	84/325	
Unadjusted	1.00 (reference)	0.83 (0.53‐1.30)	0.63 (0.43‐0.94)	.023
Model 1	1.00 (reference)	0.83 (0.53‐1.30)	0.63 (0.42‐0.95)	.025
Model 2	1.00 (reference)	0.83 (0.53‐1.30)	0.61 (0.41‐0.91)	.014
Model 3	1.00 (reference)	0.83 (0.53‐1.31)	0.61 (0.41‐0.92)	.016

a Model 1: adjusted for age, education, occupation, yearly family income.

b Model 2: adjusted for age, education, occupation, yearly family income, smoking status, drinking status, and clean the vagina.

c Model 3: adjusted for age, education, occupation, yearly family income, smoking status, drinking status, clean the vagina, vaginal pH, or hrHPV.

d CIN2+: CIN2, CIN3, and cervical cancer.

e CIN3+: CIN3 and cervical cancer.

Combining above‐mentioned results, we have investigated the mediation effects of high‐risk HPV infection on vaginal pH level and the development of CIN (Table 5). A significant mediation effect of high‐risk HPV infection was observed in the association between vaginal pH level with CIN2+ (*P* = .002) and CIN3+ (*P* = .004). The mediation proportions of high‐risk HPV infection were calculated 36.2% (95% CI: 11.4%‐71.4%) for CIN2+ and 18.1% (95% CI: 7.8%‐36.7%) for CIN3+. Furthermore, the mediation effects of high‐risk HPV infection on vaginal pH level and the development of CIN among older (age > 50) women was approximate [CIN2+, OR (95% CI) = 40.7% (4.8%‐90.4%), *P* = .026; CIN3+, OR (95% CI) = 15.9% (6.7%‐33.4%), *P* = .018]. However, no significant associations between vaginal pH level with CIN1 or CIN2 in all age women, except CIN2 in younger (age ≤ 50) women [OR (95% CI) = 33.6% (4.5%‐84.5%), *P* = .031].

**Table 5:** Mediation effects of high‐risk HPV infection on vaginal pH level and the development of CIN

Variable		Total effect OR (95% CI)	Direct effect OR (95% CI)	Mediating effect	*P*‐value
Independent	Dependent				
All women					
Vaginal pH level	CIN1	1.01 (0.99‐1.03)	1.01 (0.99‐1.03)	—	—
	CIN2	0.99 (0.96‐1.02)	1.00 (0.97‐1.03)	—	—
	CIN2+	0.98 (0.96‐1.00)	0.99 (0.97‐1.00)	36.2% (11.4%‐71.4%)	.002
	CIN3+	0.98 (0.97‐0.99)	0.98 (0.97‐0.99)	18.1% (7.8%‐36.7%)	.004
Age ≤50					
Vaginal pH level	CIN1	1.00 (0.97‐1.02)	1.00 (0.97‐1.03)	32.8% (0.0%‐100.0%)	.177
	CIN2	0.97 (0.93‐1.01)	0.98 (0.94‐1.02)	33.6% (4.5%‐84.5%)	.031
	CIN2+	0.98 (0.95‐1.01)	0.99 (0.96‐1.01)	28.7% (4.1%‐79.1%)	.056
	CIN3+	0.99 (0.98‐1.01)	0.99 (0.98‐1.01)	25.0% (1.1%‐91.2%)	.087
Age >50					
Vaginal pH level	CIN1	1.02 (1.00‐1.05)	1.02 (1.00‐1.05)	—	—
	CIN2	1.02 (0.99‐1.06)	1.03 (0.99‐1.06)	—	—
	CIN2+	0.98 (0.96‐1.01)	0.99 (0.97‐1.01)	40.7% (4.8%‐90.4%)	.026
	CIN3+	0.97 (0.95‐0.99)	0.97 (0.96‐0.99)	15.9% (6.7%‐33.4%)	.018

## 4 DISCUSSION

High‐risk HPV infection was found to be positively related to CIN risk in our large study. There was also a significant negative correlation between vaginal pH and the risk of CIN3+. Women with hrHPV infection and middle pH (4.6‐5.0) were more likely to develop CIN2+, CIN3+, than those with low pH and high pH. Results of RCS showed that CIN3+ has the highest risk when the vaginal pH is about 4.7. Moreover, subjects infected with hrHPV showed a significant mediation effect between vaginal pH level with CIN2+ and CIN3+, especially for older (>50 years) women.

The role of vaginal pH in cervical lesion pathogenesis remains unclear. The female vaginal microenvironment is dominated by lactobacilli, accounting for more than 90%.^18^, ^22^ Lactobacillus, which can convert glycogen in the vaginal epithelial cells into lactic acid, maintain the vaginal pH at 3.8~4.5, meanwhile produce hydrogen peroxide and bacteriocin, etc, to keep the stability of the vaginal microenvironment.^22^, ^23^ However, various infections, including bacterial vaginosis (BV), vulvovaginal candidiasis (VVC), trichomonas vaginitis (TV), and reduction in estrogen may cause abnormalities of the vaginal microenvironment, such as decreased lactobacilli, elevated vaginal pH, etc.^23^, ^24^, ^25^ The abnormal vaginal microenvironment, which damages the vaginal mucosa and cervical epithelium, may enhance the continuous HPV infection, reduce the clearance rate of HPV, and ultimately increased the risk of CIN.^26^, ^27^ Our results also indicated that the risk of CIN2+, CIN3+ was significantly increased when the vaginal pH > 4.5 (middle and high pH).

Persistent infection with high‐risk human papillomavirus is sufficient but not necessary to cause CIN and cervical cancer.^28^ HPV16 and HPV18 are the most common hrHPV types and are associated with approximately 70% of the incidences of CIN3 and cervical cancer in the world.^29^, ^30^ However, studies on the distribution of hrHPV genotypes in Chinese women with CIN2+ are still limited. Our previous report demonstrated that HPV16 is the most prevalent hrHPV,^29^, ^31^ followed by HPV58 and HPV33^32^ in Chinese women with CIN2+,^33^ which is different from the data of women from Western countries. Therefore, the distribution of HPV genotypes varies from country to country.

Our results show that the interaction between vaginal pH and hrHPV infection does not significantly increase the risk of CIN1. One potential explanation is that persistent hrHPV infection is necessary for CIN2+ development,^28^ while CIN1 is mostly a transient infection, in which 60% of lesions were shown to subside within 1‐2 years naturally, 30% persisted, and only 10% progressed.^34^ If the hrHPV infection in most women subsides, a high vaginal pH level will likely not increase the risk of CIN1. Another explanation is that HPV‐caused cervical lesions progress slowly to CIN2+ or cervical cancer (ie, 10‐20 years or longer), and some CIN2+ lesions may actually resolve during this period.^34^, ^35^ Our results were cross‐sectional and did not include follow‐up data; moreover, our follow‐up time remains insufficient, given the long‐term pathogenesis of CIN. Therefore, our data may not fully reflect the joint effect of vaginal pH and hrHPV on CIN1 risk.

The main limitation of this study may be its cross‐sectional nature; therefore, establishing a causal relationship was not feasible. We will continue to assess the relationship between vaginal pH and CIN risk as the study is ongoing. Moreover, the data were only from areas of Shanxi Province and did not represent the entire Chinese population.

## 5 CONCLUSION

In summary, vaginal pH significantly affects the risk of high‐stage CIN in our large‐scale population study. It may be possible to reduce the risk of cervical lesions by maintaining healthy vaginal pH levels.

## CONFLICT OF INTEREST

None.

## AUTHOR CONTRIBUTIONS

MH: designed, supervised, and guided the study; PT: conducted the statistical analysis and wrote the manuscript; and all authors: contributed to the design of the statistical analysis, interpreted data, critiqued and revised the manuscript, and read and approved the final manuscript.

## ETHICS

The study was approved by the Ethics Committee of the Second Hospital of Shanxi Medical University [No. 2013002]; all participants provided written informed consent.

## References

[1] Tsu V , Jeronimo J . Saving the world's women from cervical cancer. N Engl J Med. 2016;374(26):2509‐2511. 27355529 10.1056/NEJMp1604113

[2] Arbyn M , Weiderpass E , Bruni L , et al. Estimates of incidence and mortality of cervical cancer in 2018: a worldwide analysis. The Lancet Global health. 2019. 10.1016/S2214-109X(19)30482-6 PMC7025157 31812369

[3] Luo XM , Song L , Wu JL , et al. Analysis of the reported data of national rural cervical cancer screening project from 2012 to 2013, China. Zhonghua Yu Fang Yi Xue Za Zhi. 2016;50(4):346‐350. 27029367 10.3760/cma.j.issn.0253-9624.2016.04.012

[4] Bao HL , Zhao ZP , Zhang M , et al. The impact of five‐year Chinese rural area cervical cancer screening program on screening rate. Zhonghua yu fang yi xue za zhi. 2018;52(3):260‐264. 29973004 10.3760/cma.j.issn.0253-9624.2018.03.008

[5] Chen W , Zheng R , Baade PD , et al. Cancer statistics in China, 2015. CA Cancer J Clin. 2016;66(2):115‐132. 26808342 10.3322/caac.21338

[6] Moscicki A‐B , Schiffman M , Burchell A , et al. Updating the natural history of human papillomavirus and anogenital cancers. Vaccine. 2012;30(Suppl 5):F24‐33. 23199964 10.1016/j.vaccine.2012.05.089 PMC3700362

[7] Piersma SJ . Immunosuppressive tumor microenvironment in cervical cancer patients. Cancer Microenviron. 2011;4(3):361‐375. 21626415 10.1007/s12307-011-0066-7 PMC3234326

[8] Schiffman M , Castle PE , Jeronimo J , Rodriguez AC , Wacholder S . Human papillomavirus and cervical cancer. Lancet. 2007;370(9590):890‐907. 17826171 10.1016/S0140-6736(07)61416-0

[9] Murta E , Souza MAHD , Lombardi W , Borges LS . Aspectos epidemiológicos da infecção pelo papilomavírus humano. Jornal Brasileiro De Ginecologia. 1997;107(4):95‐99.

[10] Bosch FX , Muñoz N , de Sanjosé S , et al. Risk factors for cervical cancer in Colombia and Spain. Int J Cancer. 2010;52(5):750‐758. 10.1002/ijc.2910520514 1330934

[11] Demarco M , Lorey TS , Fetterman B , et al. Risks of CIN 2+, CIN 3+, and cancer by cytology and human papillomavirus status: the foundation of risk‐based cervical screening guidelines. J Low Genit Tract Dis. 2017;21(4):261‐267. 28953116 10.1097/LGT.0000000000000343 PMC5625966

[12] Cai‐Hong LU , Bao‐Hong LI , Xiao‐Bin LI , et al. Distribution characteristics of microorganisms in the vagina of patients with cervical cancer. J China Med Univ. 2011;40(3):267‐271.

[13] White BA , Creedon DJ , Nelson KE , Wilson BA . The vaginal microbiome in health and disease. Trends Endocrinol Metab. 2011;22(10):389‐393. 21757370 10.1016/j.tem.2011.06.001 PMC3183339

[14] Marotta F , Naito Y , Minelli E , et al. Chemopreventive effect of a probiotic preparation on the development of preneoplastic and neoplastic colonic lesions: an experimental study. Hepatogastroenterology. 2003;50(54):1914‐1918. 14696432

[15] Kitazawa H , Watanabe H , Shimosato T , Kawai Y , Itoh T , Saito T . Immunostimulatory oligonucleotide, CpG‐like motif exists in *Lactobacillus delbrueckii* ssp. bulgaricus NIAI B6. Int J Food Microbiol. 2003;85(1):11‐21. 12810267 10.1016/s0168-1605(02)00477-4

[16] Clarke MA , Rodriguez AC , Gage JC , et al. A large, population‐based study of age‐related associations between vaginal pH and human papillomavirus infection. BMC Infect Dis. 2012;12:33. 22316377 10.1186/1471-2334-12-33 PMC3292496

[17] Linhares IM , Summers PR , Larsen B , Giraldo PC , Witkin SS . Witkin SS. Contemporary perspectives on vaginal pH and lactobacilli. Am J Obstet Gynecol. 2011;204(2):120.e1‐.e5. 20832044 10.1016/j.ajog.2010.07.010

[18] Miller EA , Beasley DE , Dunn RR , Archie EA . Lactobacilli dominance and vaginal pH: why is the human vaginal microbiome unique? Front Microbiol. 2016;7:1936. 28008325 10.3389/fmicb.2016.01936 PMC5143676

[19] Weisberg E , Ayton R , Darling G , et al. Endometrial and vaginal effects of low‐dose estradiol delivered by vaginal ring or vaginal tablet. Climacteric. 2005;8(1):83‐93. 15804736 10.1080/13697130500087016

[20] Gameiro CM , Romão F , Castelo‐Branco C . Menopause and aging: changes in the immune system – a review. Maturitas. 2010;67(4):316‐320. 20813470 10.1016/j.maturitas.2010.08.003

[21] García‐Closas M , Herrero R , Bratti C , et al. Epidemiologic determinants of vaginal pH. Am J Obstet Gynecol. 1999;180(5):1060‐1066. 10329856 10.1016/s0002-9378(99)70595-8

[22] Mendling W . Vaginal microbiota. Adv Exp Med Biol. 2016;902:83‐93. 27161352 10.1007/978-3-319-31248-4_6

[23] Amabebe E , Anumba DOC . The vaginal microenvironment: the physiologic role of lactobacilli. Frontiers in medicine. 2018;5:181. 29951482 10.3389/fmed.2018.00181 PMC6008313

[24] Gillet E , Meys JFA , Verstraelen H , et al. Association between bacterial vaginosis and cervical intraepithelial neoplasia: systematic review and meta‐analysis. PLoS ONE. 2012;7(10):e45201. 23056195 10.1371/journal.pone.0045201 PMC3462776

[25] Li C , Wu M , Wang J , et al. A population‐based study on the risks of cervical lesion and human papillomavirus infection among women in Beijing, People's Republic of China. Cancer Epidemiol Biomarkers Prev. 2010;19(10):2655‐2664. 20719907 10.1158/1055-9965.EPI-10-0212

[26] Mitra A , MacIntyre DA , Marchesi JR , Lee YS , Bennett PR , Kyrgiou M . The vaginal microbiota, human papillomavirus infection and cervical intraepithelial neoplasia: what do we know and where are we going next? Microbiome. 2016;4(1):58. 27802830 10.1186/s40168-016-0203-0 PMC5088670

[27] Kyrgiou M , Mitra A , Moscicki AB . Does the vaginal microbiota play a role in the development of cervical cancer? Transl Res. 2017;179:168‐182. 27477083 10.1016/j.trsl.2016.07.004 PMC5164950

[28] Trottier H , Franco EL . The epidemiology of genital human papillomavirus infection. Vaccine. 2006;24(Suppl 1):S1‐S15. 16406226 10.1016/j.vaccine.2005.09.054

[29] Li N , Franceschi S , Howell‐Jones R , Snijders PJ , Clifford GM . Human papillomavirus type distribution in 30,848 invasive cervical cancers worldwide: variation by geographical region, histological type and year of publication. Int J Cancer. 2011;128(4):927‐935. 20473886 10.1002/ijc.25396

[30] Smith JS , Lindsay L , Hoots B , et al. Human papillomavirus type distribution in invasive cervical cancer and high‐grade cervical lesions: a meta‐analysis update. Int J Cancer. 2007;121(3):621‐632. 17405118 10.1002/ijc.22527

[31] Zhao Y , Zhao F , Hu S , et al. Multi‐center cross‐sectional study on type‐specific human papillomavirus infection among Chinese women. Zhonghua Liu Xing Bing Xue Za Zhi. 2015;36(12):1351‐1356. 26850387

[32] Zeng Z , Yang H , Li Z , et al. Prevalence and genotype distribution of HPV infection in China: analysis of 51,345 HPV genotyping results from China's largest CAP certified laboratory. J Cancer. 2016;7(9):1037‐1043. 27326245 10.7150/jca.14971 PMC4911869

[33] Wang Z , Li Z , Li J , et al. Prevalence and distribution of HPV genotypes in 1387 women with cervical intraepithelial neoplasia 2/3 in Shanxi Province, China. J Cancer. 2018;9(16):2802‐2806. 30123348 10.7150/jca.25614 PMC6096374

[34] Trimble CL , Piantadosi S , Gravitt P , et al. Spontaneous regression of high‐grade cervical dysplasia: effects of human papillomavirus type and HLA phenotype. Clin Cancer Res. 2005;11(13):4717‐4723. 16000566 10.1158/1078-0432.CCR-04-2599 PMC3132609

[35] Solé‐Sedeno JM , Mancebo G , Miralpeix E , et al. Utility of human papillomavirus genotyping in the management of low‐grade squamous intraepithelial lesions. J Low Genit Tract Dis. 2018;22(1):13‐16. 29271851 10.1097/LGT.0000000000000354

